# Oleaginous yeast as a component in fish feed

**DOI:** 10.1038/s41598-018-34232-x

**Published:** 2018-10-29

**Authors:** Johanna Blomqvist, Jana Pickova, Sarvenaz Khalili Tilami, Sabine Sampels, Nils Mikkelsen, Jule Brandenburg, Mats Sandgren, Volkmar Passoth

**Affiliations:** 1 Swedish University of Agricultural Sciences, Department of Molecular Sciences, Box 7015, S-75007 Uppsala, Sweden; 2 University of South Bohemia in České Budějovice, Faculty of Fisheries and Protection of Waters, Institute of Aquaculture and Protection of Waters, Husova tř. 458/102, 370 05 České Budějovice, Czech Republic; 3 Present Address: Faculty of Science and Technology, Norwegian University of Life Sciences, Ås, Norway

## Abstract

This study investigates the replacement of vegetable oil (VO) in aquaculture feed for Arctic char (*Salvelinus alpinus*) with oil produced by the oleaginous yeast *Lipomyces starkeyi* grown in lignocellulose (wheat straw) hydrolysate. VO is extensively used to partially replace fish oil in aquaculture feed, which can be seen as non-sustainable. VO itself is becoming a limited resource. Plant oils are used in many different applications, including food, feed and biodiesel. Its replacement in non-food applications is desirable. For this purpose, yeast cells containing 43% lipids per g dry weight were mechanically disrupted and incorporated into the fish feed. There were no significant differences in this pilot study, regarding weight and length gain, feed conversion ratio, specific growth rate, condition factor and hepatosomatic index between the control and the yeast oil fed group. Fatty and amino acid composition of diet from both groups was comparable. Our results in fish demonstrate that it is possible to replace VO by yeast oil produced from lignocellulose, which may broaden the range of raw materials for food production and add value to residual products of agriculture and forestry.

## Introduction

Fish is one of the most traded food commodities and has great potential to contribute to food security for a growing population^1^. Fish is already the major source of protein in many cultures^2^. Moreover, it is also the major resource of n-3 long chain polyunsaturated fatty acids (LCPUFA)^3^. Hence, aquaculture is a rapidly growing industry and is an important source of animal-based foods. This growth generates an increased demand for feed for farmed fish. Currently, fish meal and fish oil are still the primary resources to meet the demand for protein and lipids of farmed fish^2^. Aquaculture consumes about 70% of the globally produced fish oil (FO), and 90% of this oil is derived from reduction fisheries^3^. Thus, a sustainable further expansion of the aquaculture industry can only happen when alternative resources/replacements for FO can be found. Those alternatives can be both vegetable oil (VO) and terrestrial animal oil^3–6^. Although both VOs and animal fat do not provide a good supply of n-3 long chain polyunsaturated FA (LCPUFA), they are metabolised by the fish in beta-oxidation, to provide energy. It has been shown that FO can be replaced by VO or animal fat without negatively impacting fish health or growth^4–8^. In Europe, VO is the most common partial substitute for fish oil, whereas in other parts of the world, terrestrial animal fats are also incorporated into aquafeeds. Nevertheless, VOs have a broad range of applications, including direct food production and biodiesel production. Especially with a view towards the latter, discussions about the sustainability of VO production have been raised. Finding alternatives to VO may lessen the push towards monocultures, with risk for land use changes and rainforest cutting, and in general, reduce the food carbon print of aquaculture^9–13^.

Microbial oils or single cell oils have been regarded as a potential replacement for VO in biodiesel production, and in some cases even for food purposes. Oleaginous yeasts, *i.e*. yeasts that can accumulate 20% and more of their biomass as lipids, can form single cell oils from a variety of low value substrates, including lignocellulose hydrolysate^14–16^.

While there are a number of reports on utilising yeasts as a protein source in fish feed (e.g.^17,18^), only little is known about utilising yeast-derived oil in fish feed. Several oleaginous yeast species can utilise lignocellulosic hydrolysates and convert them to lipids. We have recently demonstrated that the oleaginous yeast *Lipomyces starkeyi* can efficiently synthesise lipids from the hemicellulose fraction of birch wood and the cellulose fraction of wheat straw^19,20^, and other studies have also used lignocellulose hydrolysate as a substrate for oil production with this yeast^21–24^. The lipid composition of *L. starkeyi* was shown to be similar to that of saturated fatty acids (SFA) rich VO, for example olive oil or palm oil^19,20,25^. In this pilot study, the aim was to test whether it is possible to replace VO in the feed for Arctic char (*Salvelinus alpinus*) with single cell oils derived from *L. starkeyi* grown on lignocellulose hydrolysate from wheat straw, *i.e*. a non-edible, residual material.

## Results

### Hydrolysate analysis

The cellulosic hydrolysate from wheat straw (*i.e*. the enzymatically hydrolysed solid phase after steam explosion (see method part)) contained glucose 87.3 g/l, xylose 22.2 g/l and acetic acid 3.8 g/l. Due to the high acetic acid concentration we started the fermentation with 50% hydrolysate and then pumped in 100% hydrolysate in the feeding phase of the cultivation.

### Yeast cultivation

At harvest, *L. starkeyi* cells had consumed all carbon sources, and the total yeast dry weight of 575 g (cells from four fermentors) was produced from a total amount of 23.2 l hydrolysate, *i.e*. 2628.6 g carbon sources (glucose, xylose, and acetic acid). The final intracellular lipid content of the yeast was determined to be 43 ± 0.8%, thus the total amount of yeast lipids produced was 247.25 g, corresponding to a lipid yield of 0.09. Yeast growth and carbon source consumption are illustrated in Supplementary Fig. S1. Cells were disrupted by French press, as described in Methods; successful disruption was confirmed by microscopic inspection of the cell lysate. No further oil extraction was performed, to avoid contamination with toxic solvents and to retain the yeast proteins and polysaccharides in the hydrolysate.

### Fish performance

Fish were fed with a standard experimental diet^26^ (see Methods) containing standard ingredients and either VO and casein (control diet) or disrupted *L. starkeyi* cells instead of VO and casein. The fatty acid composition of the feeds is shown in Table 1. In both feeds, the main source of amino acids was fish meal. Accordingly, the amino acid profiles of the control- and yeast-based feeds did not differ significantly (Table 2). Initial and final weight and length, liver weight and the calculated performance factors are presented in Table 3. Initial weight of fishes was 148.2 g (control) and 149.8 g (yeast oil feeding), and the final weight was 265 g in both cases. There were no significant differences between the control and yeast fed fish regarding feed conversion rate (FCR), specific growth rate (SGR), condition factor (CF) and hepatosomatic index (HSI), indicating that both feeds were metabolised in a similar way and the addition of yeast in the feed did not negatively impact growth.

**Table 1:** Fatty acid composition (% of total FA) of the two experimental diets (duplicate analyses, the deviation of the single measurements was below 1.5%).

Fatty acid	Proportion in VO (control) feed [% of total fatty acids]	Proportion in yeast oil feed [% of total fatty acids]
C14:0	3.5	4.5
C16:0	15.9	21.3
C18:0	4.0	3.6
C18:1, tot	30.2	26.0
C20:1, tot	1.5	2.0
C22:1, tot	4.2	4.2
C18:2n-6	3.8	2.8
C20:4n-6	0.45	0.53
C18:3n-3	1.4	1.7
C20:5n-3	8.5	9.5
C22:5n-3	1.0	1.0
C22:6n-3	6.8	8.1

**Table 2:** Amino acid composition of the two experimental diets.

Amino acid	Proportion of total determined amino acids [%] in VO feed^a^	Proportion of total determined amino acids [%] in yeast oil feed^a^
Alanine	6.4	6.8
Arginine	6.6	6.7
Aspartic acid	9.4	9.6
Cysteine + Cystine	1.0	1.0
Glutamic acid	16.7	16.2
Glycine	6.6	7.1
Histidine	2.1	2.1
Isoleucine	4.2	4.2
Leucine	8.0	8.0
Lysine	7.7	7.5
Methionine	2.8	3.0
Phenylalanine	4.3	4.3
Proline	5.6	5.5
Serine	4.8	4.8
Threonine	4.4	4.5
Tyrosine	3.7	3.7
Valine	5.1	5.1

^a^Amino acid analyses were performed by Eurofins Food & Feed Testing Sweden AB. The confidence interval of all values is 15%.

**Table 3:** Performance factors for fish fed with either control feed or feed with yeast as a substitute for VO.

	Control (n = 24)	Yeast (n = 24)
Initial length (cm)	23.63 ± 0.05	23.58 ± 0.30
Initial weight (g)	148.2 ± 3.9	149.8 ± 4.4
Final length (cm)	27.79 ± 0.76	27.91 ± 0.79
Final weight (g)	265.1 ± 34.7	265.0 ± 29.8
Liver weight (g)	4.15 ± 0.88	4.00 ± 0.59
FCR^*^ (%)	1.86 ± 0.55	1.69 ± 0.38
SGR^*^ (%)	0.95 ± 0.18	1.00 ± 0.14
CF^*^ (%)	1.17 ± 0.06	1.16 ± 0.02
HSI^*^ (%)	1.47 ± 0.11	1.42 ± 0.06

Data are presented as means ± standard deviation. Feeding trial was conducted in triplicates with n = 8 in each tank (n total = 24 fish in each treatment). No significant differences between the feeds were identified.

^*^Abbreviations: FCR- feed conversion rate, SGR- specific growth rate, CF- condition factor, HSI- hepatosomatic index.

There was a large standard deviation of the individual fish weight, both in the yeast-fed treatment and the control towards the end of the experiment. This effect was most likely due to the small number of fish, which enabled a few dominant individuals to consume a major proportion of the provided feed, at the costs of other, minor individuals, which hardly showed any growth. The number of fishes was adjusted to the size of the 3 tanks and 2 months feeding to ensure appropriate water parameters such as NH_4_^+^ and oxygen tension when fish biomass increases. However, the total mass of the fish did not significantly differ, in spite of the dominant individuals. Consequently, sampling was carried out on fishes representing all sizes from all units. This growth effect does not hinder the evaluation of the fatty acid composition of the tissue samples, as the individual fish reflected the feed fatty acid profile, which is a common result in experiments performed on salmonids.

### Lipid content and fatty acid composition

The total weight (whole fish), fat content and fatty acid profile of the muscle tissue of six yeast fed fishes and six control fed fishes are shown in Table 4 (two from each tank).

**Table 4:** Weight, fat content and fatty acid profile in the fillet (dark and light muscle tissues) from Arctic char fed with either control feed or feed with yeast as a substitute for VO.

	Control, n = 6	Yeast, n = 6
Weight, g	271 ± 51.0	295 ± 36.1
Fat content %	4.70 ± 0.64	7.07 ± 2.81
**Fatty acid composition**		
C14:0	4.10 ± 0.20	4.08 ± 0.23
C15:0	0.29 ± 0	0.30 ± 0.01
C16:0	16.0 ± 0.41	17.6 ± 0.64
C17:0	0.36 ± 0.03	0.33 ± 0.04
C18:0	2.51 ± 0.24	2.43 ± 0.15
C20:0	1.23 ± 0.04	1.30 ± 0.16
C16:1n-7	6.04 ± 0.35	6.76 ± 0.61
C18:1n-9	31.9 ± 0.33	31.0 ± 0.81
C20:1n-9	3.19 ± 0.03	3.13 ± 0.31
C22:1n-9	2.29 ± 0.07	2.29 ± 0.20
C18:2n-6	5.82 ± 0.08^a^	4.78 ± 0.50^b^
C20:2n-6	0.16 ± 0.02	0.16 ± 0.04
C20:4n-6	0.39 ± 0.01	0.40 ± 0.05
C18:3n-3	1.24 ± 0.08	1.09 ± 0.16
C20:5n-3	6.98 ± 0.37	7.15 ± 0.44
C22:5n-3	1.38 ± 0.06	1.46 ± 0.13
C22:6n-3	14.3 ± 1.02	13.8 ± 2.25
SFA*	24.5 ± 0.85	26.0 ± 0.58
MUFA*	41.3 ± 0.41	41.1 ± 1.67
PUFA*	30.4 ± 0.93	29.0 ± 2.04
n-3	23.9 ± 0.87	23.5 ± 2.53
n-6	6.48 ± 0.08^a^	5.48 ± 0.50^b^
n-6/n-3	0.27 ± 0.01	0.24 ± 0.05

The different letters above the numbers represents values with significant differences; without letters represents values with no significant differences. (n = 6, Mean ± standard deviation). ^*^SFA = saturated fatty acids, MUFA = mono unsaturated fatty acids, PUFA = poly unsaturated fatty acids.

Overall, no significant differences between the two different feeds were observed, except for linoleic acid (C18:2 n-6) where the fish fed with control feed had slightly higher levels compared to yeast fed fish.

## Discussion

In this study, we investigated whether it is possible to replace VO with oil produced by an oleaginous yeast, *L. starkeyi*, grown on lignocellulose (wheat straw) hydrolysate. Inclusion of yeast oil into fish feed has been tested previously but in the context of replacing fish oil in the feed, using oil from genetically engineered *Yarrowia lipolytica* cultivated on first generation substrate (glucose)^27^.

Our study shows that it is possible to convert second generation substrate (lignocellulose) to a feed component, enabling the replacement of feed oil (mostly VO) as an energy source in aquaculture. VOs are listed among the products causing the largest environmental impacts. They are also regarded as the fastest growing food commodities worldwide^28^. Some vegetable oils have a high greenhouse gas potential associated with their production: for instance palm- and soybean oil are estimated to emit more than 2000 kg CO_2_ equivalents per ton produced, and considerable areas of arable land are used for producing vegetable oils^29^. Since biodiesel is also produced from vegetable oils, their consumption in the EU greatly exceeds local production, and thus, a major proportion of the utilised plant oil has to be imported^30^. There are reports of rainforest clearing due to palm- and soya oil production and there are moves in the EU to reduce the use of imported vegetable oils, especially palm oil (http://www.europarl.europa.eu/sides/getDoc.do?pubRef=-//EP//TEXT+REPORT+A8-2017-0066+0+DOC+XML+V0//EN).

The yeast cells contain, apart from oil, also proteins and other components that can be utilised by the fish. The first implication of this is that the yeast cells contributed to protein biomass in the feed; this was adjusted by removing the casein from the yeast feed, whereas it was the standard protein additive in the control feed, as commonly used in other fish feeding trials^26^. The second implication is that it was not necessary to extract the oil from the mechanically disrupted yeast cells. This is advantageous compared to for instance microbial biodiesel production, where extraction is regarded as one of the most crucial steps in obtaining a sustainable process^31^. Analyses of growth parameters and composition of the final fish demonstrated that there was no negative impact of replacing VO and casein by *L. starkeyi*-biomass. The amino acid profile of the yeast-based feed did not change compared to the control. There was a slight but significant decrease in the total amount of n-6 fatty acids in the yeast fed fish. A low n-6/n-3 ratio is advantageous, as in most modern diets this ratio is too high, leading to a variety of diseases^32^. Our experiment demonstrates that it is possible to replace terrestrial plant- and animal based lipid and protein sources by yeast biomass. The fatty acid composition of yeast strains varies with both strain and cultivation conditions^19,20,33^. Selecting appropriate yeast strains and culture conditions may thus represent a possibility to positively influence the fatty acid composition and thereby the n-6/n-3 ratio.

From the lignocellulose substrate, 0.09 g lipids were produced per g consumed carbon source. This is within the range of values previously reported in similar cultivations^19,20,34^. In this study, yeast cultivation was performed to generate biomass for the fish trial; optimisation of the yeast fermentation conditions was not within the scope of the study. Nevertheless, rapid and efficient lipid production from the substrate can greatly improve the overall energy output and greenhouse gas impacts of any single cell oil production process^31,35^, and therefore, optimisation of fermentation conditions and strains for lipid production is one of the major topics of our ongoing research.

This pilot study, to the best of our knowledge, investigates for the first time the utilisation of lignocellulose-derived yeast oil in fish feed. The results demonstrate that it is possible to completely replace VO and partially replace protein (casein in the control feed, in the present study) with the yeast biomass, without any significant effects on fish growth and final quality. Previous studies have shown that there is a limit to including yeast-based protein into fish diets^17,27^. On the other hand, there are also studies indicating a positive effect of yeast cell wall β-glucan on the immune system of fish^36^ and a barrier function of yeasts against prions^37^. Moreover, utilising different yeast strains and different lignocellulose substrates may have some impact on the final quality of the fish. All these possible effects require further investigation and will be the subject of future studies.

## Methods

### Strains and media

*L. starkeyi* CBS 1807 (Centraalbureau vor Schimmelcultures, Utrecht, The Netherlands) was maintained on YM-agar plates (glucose 10 g/l, yeast extract 3 g/l, peptone 5 g/l, malt extract 3 g/l, Agar 16 g/l). The pre-culture medium was YPD (glucose 20 g/l, yeast extract 10 g/l, peptone 20 g/l).

### Preparation of hydrolysate

The steam explosion and enzymatic hydrolysis was performed at the Department of Chemical Engineering, Lund University, Sweden. Wheat straw was soaked with 1% acetic acid over night, and fluid removed by pressing. The acid soaked biomass was then steam exploded at 190 °C for 10 min in a 10 L steam pretreatment reactor. The liquid fraction (mainly hemicellulose) was separated from the solid fraction and the latter was enzymatically hydrolysed. The hydrolysis was performed at 45 °C and pH 4.8. Cellic CTec3 enzyme cocktail (Novozyme A/S, Bagsværd, Denmark) was added at 10 FPU/g substrate. After hydrolysis, the suspension was centrifuged to separate the solid residues (mainly lignin) and repeatedly filtered, using filters with decreasing pore size in each step. The last filtration step was performed with a 0.45 μm sterile filter. The sugar and acetic acid concentration was determined by HPLC as described previously^19^.

### Pre cultures

Before inoculation in fermentors, *L. starkeyi* was cultivated in two steps with increasing medium volumes. For the first pre-culture, a loopful of *L. starkeyi* cells was inoculated from a YM-agar plate into 100 ml YPD-medium in 500 ml baffled shake flasks and incubated at 25 °C and 150 rpm in a rotatory shaker. After 48 h, the 100 ml culture was transferred to 400 ml YPD medium in a 3 l shake flask and incubated at 25 °C and 150 rpm for 72 h. The cells were harvested by centrifugation (4000 g, 10 min) and washed twice with saline solution (NaCl, 9 g/l). After washing, the pellet was resuspended in 50 ml saline and inoculated into the fermentor.

### Fed-batch cultivation in fermentors

*L. starkeyi* was cultivated in four Dolly fermentors (Belach Bioteknik, Stockholm, Sweden, working volume 8 l) at 25 °C. A volume of 1.5 l of sterile filtered cellulose hydrolysate was added to each fermentor containing 1.5 l sterile deionised water, representing a starting volume of 3 l comprised of 50% cellulose hydrolysate. The pH was set at 5 and automatically controlled by addition of NaOH (25% w/w) or 3 M H_3_PO_4_. The aeration was initially 1 l/min; during the experiment it was continuously increased up to 5 l/min. The dissolved oxygen tension (pO2) was controlled by a DO-electrode, set to 20% and maintained by changing the stirring speed. One ml of polypropylene glycol 2000 (Alfa Aesar, Karlsruhe, Germany) was added to prevent foaming. *L. starkeyi* was first cultivated in a batch phase for 48 h, then the fed-batch phase started with pumping cellulosic hydrolysate at a speed of approx 24 ml/h in 7.5 days, *i.e*. 4.3 l of hydrolysate was added to each fermenter during the feeding phase; the total amount of hydrolysate was thus 5.8 l per fermenter.

### *L. starkeyi* harvesting

Cells were centrifuged at 5400 g for 10 min, washed with deionised water and then disrupted in a French press (Constant systems LTD, Daventry, UK) at 40 psi. Dry weight of the disrupted cells was determined by drying a portion of the cell-lysate in a Precisa xm 60 oven (Precisa Instruments LTD, Dietikon, Switzerland) and the disrupted cells were stored at −20 °C until incorporation into the fish feed^18^.

### Fish feed preparation

The composition of the fish feed is shown in Table 5 ^26^. The ingredients were mixed by hand to a homogeneous consistency and pressed through a kitchen meat grinder. The feed was dried at room temperature for 48 h before vacuum packing into air tight plastic bags. Total amino acids were quantified in the prepared feeds (Eurofins Food & Feed Testing Sweden AB, Lidköping, Sweden, Method: SS-EN ISO 13903:2005).

**Table 5:** Ingredients in the two types of fish feed: the control feed and the feed with yeast as a substitute for VO.

Feed ingredients	Control feed		Yeast feed	
	(g)	%	(g)	%
Fish meal	550	49.4	550	50.3
Fish oil	130	11.7	130	11.9
VO (Olive oil)	55	4.94	0	0
Mineral and vitamin mix	4	0.36	4	0.37
Wheat meal	295	26.5	245	22.4
Casein	55	4.94	0	0
Ca_2_SO_4_	25	2.24	25	2.29
Yeast DM	0	0	140	12.8
Total	1114	100	1094	100

### Feeding trial

The experiment was carried out in accordance with EU legislation (*i.e*., Directive 2010/63/EU), and received the approval of the Ethical Committee for Animal Experiments in Umeå, Sweden.

Arctic char was kept in flow through system with natural photo period at Kälarne Aquaculture North, Sweden. The water temperature was ambient, approx 12 °C and the water system was always fully aerated from the inlet. Inlet water quality was always assured. The tanks were 1 × 1 m and water depth 20 cm. Tanks were randomly assigned to the two diets with randomly selected fish (n = 8/tank). Prior to the trial all fish were fed a commercial diet suitable for Arctic char juveniles. The feed was distributed by band feeders 4 times a day^26^. Fish was anaestesised before handling^38^.

The Arctic char were measured for weight and length and then divided into six tanks (n = 8): three were fed with the control feed and three with yeast feed. Feeding ratio was 2% of the actual biomass in the tanks.

After 2 months, the fish were weighed and measured again after a 24 h starvation period and liver weight was registered. After filleting, the muscle tissue was frozen on dry ice and then stored at −80 °C until lipid extraction.

### Fish performance

Based on the measurements and the consumed feed, feed conversion ratio (FCR), specific growth rate (SGR), condition factor (CF) and hepatosomatic index (HSI) were calculated as follows:

$${\rm{FCR}}={\rm{F}}/({\rm{Wt}}-{\rm{W}}0)$$

$${\rm{SGR}}=[(\mathrm{ln}\,{\rm{Wt}}-\,\mathrm{ln}\,{\rm{W}}0)/{\rm{t}}]\times 100$$

$${\rm{CF}}={\rm{Wt}}/{{\rm{TL}}}^{3}\times 100$$

$${\rm{HSI}}=({\rm{Wl}}/{\rm{Tw}})\times 100$$

where Wt = final weight of fish in g; W0 = initial weight of fish in g; F = amount of dry feed fed in g; t = time (days); TL = total length in cm, Wl = weight of liver in g; Tw = total weight of fish without liver in g.

### Lipid extraction from yeast, feed and fish

The lipid content of yeast cells was determined as previously described^19,20^. For the fish and feed, total lipid analysis was performed according to Pettersson, *et al*.^26^. Lipids were extracted from six muscle samples from each treatment (sourced from two fish from each replicate) and from the feeds. A subsample of 1 g of fish feed or muscle (light and dark) of individual Arctic char was used for lipid extraction (in duplicate). The sample was homogenised in hexane:isopropanol (HIP; 3:2, v-v) with an Ultra-Turrax (Janke and Kunkel, IKA Werke, Staufen, Germany). For lipid and non-lipid phase separation, 6.67% of Na_2_SO_4_ was added to the homogenate and it was centrifuged. After gravimetrical identification of the total lipid content from dried samples, the lipids were stored in hexane at −80 °C for further analysis. All chemicals and solvents (reagent grade) were purchased from Merck (Darmstadt, Germany) except chloroform (Sigma Chemicals Co. St. Louis, MO, USA). The solvents were used without further purification.

### Determination of fatty acid profiles

Fatty acid methyl esters (FAME) from total lipids in muscle and feeds were prepared with BF_3_ methanol according to the method described by Appelqvist^39^. FAME were stored in hexane at −80 °C for further analysis.

FAME were analysed by GC using a CP 3800 instrument (Varian AB, Stockholm, Sweden) equipped with a flame ionization detector and a split injector, and separated on a 50 m fused silica capillary column BPX 70 (SGE, Austin, Tex) (0.22 mm i.d × 0.25 µm film thickness)^40^. The injector temperature was 230 °C and the detector temperature 250 °C. Helium was the carrier gas, at a flow rate of 0.8 mL/min, and nitrogen was used as make-up gas. Peaks were identified by comparing their retention times with those of the standard mixture GLC 68A (Nu-check Prep, Elysian, USA) and quantified using an internal standard (methyl-15-methylheptadecanoate; Larodan Fine Chemicals AB, Malmö, Sweden). Peak areas were integrated using Galaxie chromatography data system software version 1.9 (Varian AB, Stockholm, Sweden).

### Statistics and calculations

Mean values, standard deviations and FA percentages were calculated in Excel and statistical analyses were performed using the Statistica CZ 12 software package. One-way analysis of variance (ANOVA) and Tukey’s HSD test were performed to characterise the differences between control and experimental group. The performance factors data were treated by One-way ANOVA in Excel.

## Electronic supplementary material

Supplementary Figure S1

## Data Availability

The datasets generated and analysed during the current study are available from the corresponding author upon request.
