# Supplementary material for: Oleaginous yeast as a component in fish feed
Source: Sci Rep. 2018 Oct 29;8:15945. doi: 10.1038/s41598-018-34232-x (PMC6206134; doi:10.1038/s41598-018-34232-x)
Supplement: Supplementary file 1 — Supplementary Figure S1 [file 41598_2018_34232_MOESM1_ESM.pdf]

Oleaginous yeast as a component in fish feed

Johanna Blomqvist, Jana Pickova, Sarvenaz Khalili Tilami, Sabine Sampels, Nils Mikkelsen, Jule Brandenburg, Mats Sandgren, Volkmar Passoth

Figure S1

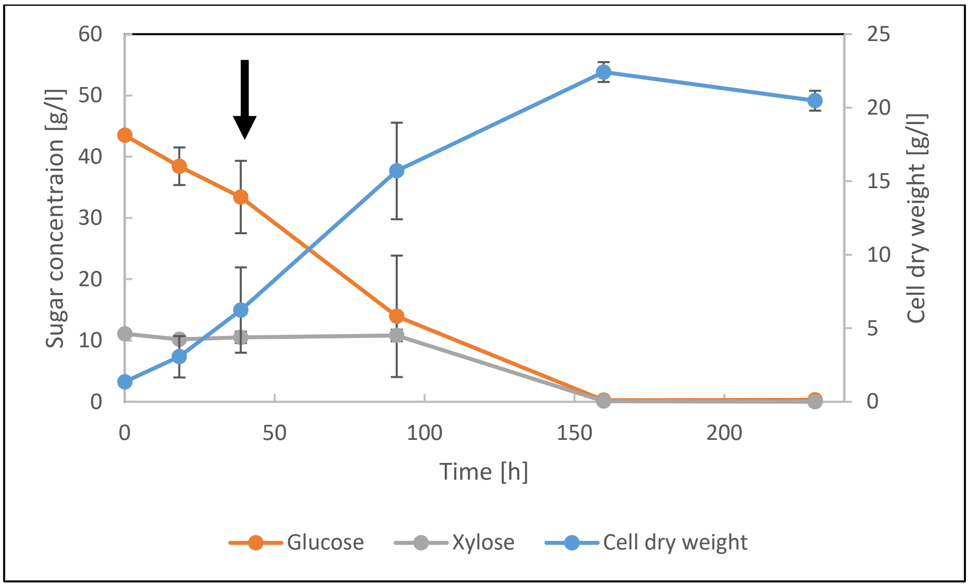

Figure S1. Growth and sugar consumption of *Lipomyces starkeyi* in wheat straw hydrolysate. The fermentation was started in 50% hydrolysate, after 48 h (indicated by the black arrow) feeding of non-diluted hydrolysate started (feeding rate 24 ml/h). The figure shows average values and standard deviations of four independent fermentations.
